# Novel miR-122 delivery system based on MS2 virus like particle surface displaying cell-penetrating peptide TAT for hepatocellular carcinoma

**DOI:** 10.18632/oncotarget.10681

**Published:** 2016-07-18

**Authors:** Guojing Wang, Tingting Jia, Xixia Xu, Le Chang, Rui Zhang, Yu Fu, Yulong Li, Xin Yang, Kuo Zhang, Guigao Lin, Yanxi Han, Jinming Li

**Affiliations:** ^1^ National Center for Clinical Laboratories, Beijing Hospital, National Center of Gerontology, Beijing, People's Republic of China; ^2^ Beijing Engineering Research Center of Laboratory Medicine, Beijing Hospital, Beijing, People's Republic of China; ^3^ Central Research Laboratory, Peking Union Medical College Hospital, Chinese Academy of Medical Sciences and Peking Union Medical College, Beijing, People's Republic of China; ^4^ Graduate School, Peking Union Medical College, Chinese Academy of Medical Sciences, Beijing People's Republic of China; ^5^ Department of Clinical Laboratory, Beijing Chaoyang Hospital, Capital Medical University, Beijing, People's Republic of China

**Keywords:** MS2 VLP, phage surface display, TAT peptide, miR-122

## Abstract

Current treatments for hepatocellular carcinoma (HCC) have shown inadequate. MicroRNA-122 (miR-122) mediated RNA interference brings new prospects. A safe, efficient miRNA delivery system is an indispensable assurance. Previously, we developed an MS2 bacteriophage virus-like particle (VLP)-based microRNA delivery system crosslinked with the HIV TAT peptide, which served as an effective inhibitor in the treatments of systemic lupus erythematosus and osteoporosis. However, defects, such as low crosslinking efficiency, high cost, and potential toxicity of the crosslinking agent, needed to be confronted. Therefore, TAT peptide was designed to display on the surface of MS2 VLPs, instead of being chemically crosslinked, using the platform of phage surface display. The results reflected that MS2 VLPs displaying TAT could effectively penetrate the cytomembrane and deliver miR-122. Additionally, its inhibitory effects on HCC were significant in Hep3B, HepG2, and Huh7 cells and Hep3B related animal models. Thus, we have established a novel miR-122 delivery system based on MS2 VLPs surface displaying TAT peptide, which could effectively perform the function of penetrating cytomembrane and the inhibition of HCC.

## INTRODUCTION

Hepatocellular carcinoma (HCC) is one of the most prevalent malignant tumor, with the third most frequent cause of cancer-related deaths [1–3]. Considering the complexity of its pathogenesis, high metastasis, and poor prognosis [4–6], current therapies have reflected respective imperfections and limitations. Hence, new potential HCC treatments are required.

MicroRNAs (miRNAs), endogenous small non-coding RNAs, can interact with target messenger RNA (mRNA) 3′-untranslated regions (3′-UTR) and negatively regulate their gene expression by inhibiting translation or degrading targeted mRNAs at the post-transcriptional level [7]. Because the potent therapeutic effects of miRNA interference have been confirmed in cancer [7–9], using specific miRNAs to treat HCC is now a possible approach. MRX34 as the first anticancer miRNA drug has entered Phase I clinical trials in patients with primary liver cancer and metastatic HCC [10–12], which suggested the prospects of applying therapeutic miRNAs to HCC. miR-122, the downregulation of which has been identified both in patients with HCC and HCC-derived cell lines [13–16], negatively regulates several target gene expressions, such as those of cyclin G1, serum response factor (Srf), and insulin-like growth factor 1 receptor (Igf1r). Moreover, the functional mechanism [17–20] and several targets of miR-122 have been clearly revealed which facilitate further research. The miR-122 mimetic was delivered to observe its inhibitory effects on HCC in vivo and in vitro, reflecting its potential as an anticancer miRNA drug candidate.

miRNA delivery depends primarily on an effective transport system [21, 22]. However, the drawbacks of existing miRNA delivery carriers, such as cytotoxicity [23], limited transduction efficiency [24], and potential carcinogenicity [25], remain hard to overcome. MS2 bacteriophage virus-like particles (MS2 VLPs) harboring specific RNA fragments possess favorable features, such as stability, biocompatibility, biodegradability, and suitable molecular size [26–29], making them appropriate miRNA vectors.

Previously, we developed an MS2 VLP-based microRNA-146a delivery system and conjugated human immunodeficiency virus (HIV) TAT (47-57) cell-penetrating peptide to it, using chemical cross-linkers [30]. MS2-miR-146a VLPs could effectively eliminate autoantibodies thus ameliorating systemic lupus erythematosus (SLE) progression in lupus-prone mice [31] and suppress osteoclast differentiation [32]. MS2 VLPs also encapsulated maternally expressed gene 3 long non-coding RNA (MEG3 lncRNA), and were crosslinked with the GE11 polypeptide on the surface, proving beneficial to cancer therapy [33]. Nevertheless, the prominent problems of low crosslinking efficiency and large inter-assay variations, in preparation of MS2 VLP crosslinking peptides, cannot be neglected, because of random combinations being generated during the process. Other issues, such as the potential toxicity of the crosslinking agent, long preparation process, and tedious procedures, should also be addressed.

The technique of phage surface display brings new prospects. Previously, the immunogenic epitope peptides were successfully displayed by MS2 VLP platforms, which elicited broadly cross-reactive and cross-neutralizing antibodies against diverse human papillomavirus (HPV) types [34–36]. Nevertheless, the related reports referring to the cell penetrating peptides displayed on the surface of MS2 VLPs, which played the delivery role, had not been discovered. Through this study, we intend to improve the MS2 VLP-based microRNA delivery system combined with phage surface display platform. It is crucial to select appropriate cell-penetrating peptides.

HIV TAT was the first reported cell penetrating peptide [37]. The regions of TAT 47-57 belong to the protein transduction domain and possess powerful, nontoxic, and efficient transport capacity, which had been clearly confirmed [38–40]. Hence, TAT 47-57 was selected and designed to display on the surface of MS2 VLP, instead of chemical crosslinking. Here, the cell-penetrating function of MS2 VLPs displaying TAT peptide was further identified, and its inhibitory effect on HCC, mediated by VLPs encapsulating miR-122, would be discovered.

## RESULTS

### Preparation and identification of MS2 VLPs displaying TAT

**Figure 1:**
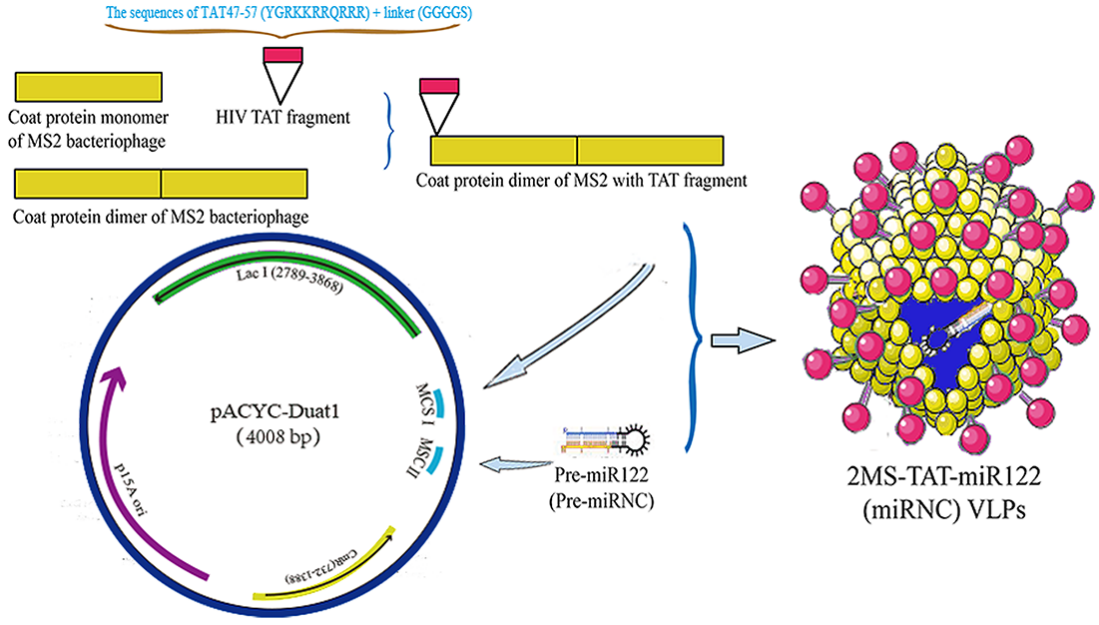
The schematic diagram of construction and preparation of 2MS-TAT-miR122 VLPs

**Figure 2:**
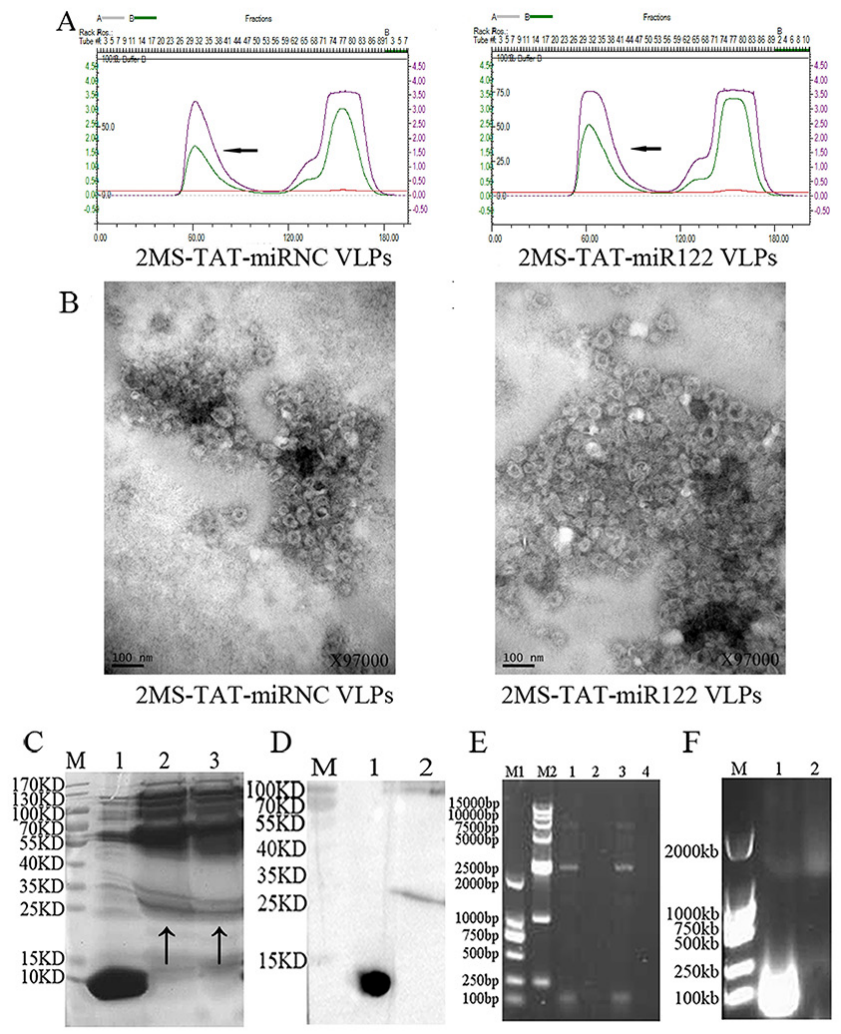
Identification of 2MS-TAT-miR122 VLPs and its negative control **A.** Purification of VLPs. The peak of the target protein is marked by an arrow. Left panel, 2MS-TAT-miRNC VLPs; right panel, 2MS-TAT-miR122 VLPs. **B.** Verification of VLPs by TEM. Left panel, 2MS-TAT-miRNC VLPs; right panel, 2MS-TAT-miR122 VLPs. **C.** Verification of VLPs by SDS-PAGE. Lane M, molecular mass marker; Lane 1, wild-type MS2 VLPs; Lane 2, 2MS-TAT-miRNC VLPs; Lane 3, 2MS-TAT-miR122 VLPs; The target protein is marked by an arrow. **D.** Verification of VLPs by Western blot. Lane M, molecular mass marker; Lane 1, wild-type MS2 VLPs; Lane 2, 2MS-TAT-miR122 VLPs. **E.** RT-PCR detection of miRNA packaged by the VLPs. Lane M1, DL2000; Lane M2, DL15000; Lane 1, 2MS-TAT-miRNC VLPs; Lane 2, negative control; Lane 3, 2MS-TAT-miR122 VLPs; Lanes 4, negative control. **F.** Nuclease resistance assay of VLPs. The MS2 VLPs displaying TAT in Lane 2 was incubated with DNase I and RNase A, but those in Lanes 1 was not.

According to the previous report [34–36], the TAT peptide was connected to the amino-terminus of the dimer of the MS2 coat protein, whereas the pre-miR122 (negative control: pre-miRNC) was designed with a pac site to facilitate packaging (Figure 1). After a series of preparation steps, the target protein was harvested after about 60 minutes (Figure 2A).

Further identifications were performed as follows. First, the assembly effects of VLPs surface displaying TAT were confirmed by transmission electron microscopy (TEM, JEM-1230, Japan) (Figure 2B). The size of 2MS-TAT-miRNA VLPs was slightly larger in diameter than wild-type MS2 VLPs (Supplementary Figure S1B). Moreover, the appearance of MS2 VLPs displaying TAT was less regular than of wild-type MS2 VLPs. Second, sodium dodecyl sulfate polyacrylamide gel electrophoresis (SDS-PAGE) results revealed that the dimer of the MS2 coat protein was approximately 25-35 kDa (Figure 2C), while that of wild-type MS2 VLPs was nearly 10-15 kDa. The results of western blot analysis with anti-MS2 antibody further indicated the position of MS2 coat protein on SDS-PAGE (Figure 2D). Third, the miRNAs encapsulated in the 2MS-TAT-miRNA VLPs were extracted and identified by reverse transcription polymerase chain reaction (RT-PCR), which indicated that both 2MS-TAT-miR122 VLPs and 2MS-TAT-miRNC VLPs encapsulated approximately 100 bp miRNAs sequences (Figure 2E). Finally, a band of 1000-2000 bp on agarose gel electrophoresis with GelRed staining, was observed after 2MS-TAT-miRNA VLPs were digested with DNase I and RNase A (Figure 2F), which suggested that the miRNAs harbored by MS2 VLPs displaying TAT were nuclease-resistant.

### Cell-penetrating ability of 2MS-TAT-miRNA VLPs

**Figure 3:**
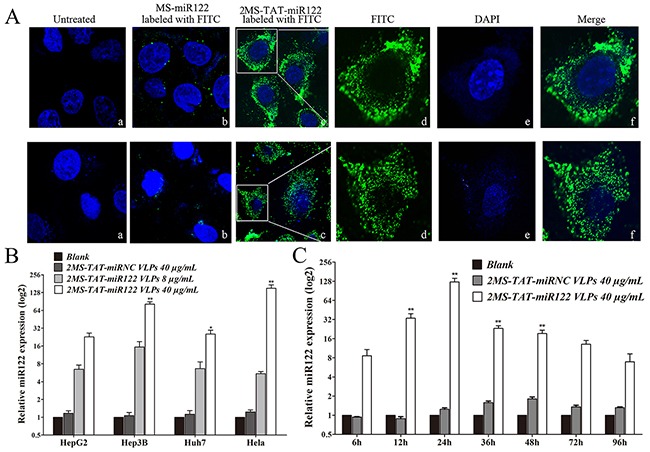
The capabilities for penetrating cytomembrane and miR-122 delivery of MS2 VLP displaying TAT **A. Upper panel**, Penetrating cytomembrane of 2MS-TAT-miR122 VLPs in Hep3B cell. a, untreated cells; b, Hep3B treated with MS-miR122 VLPs labeled with FITC; c: Hep3B treated with 2MS-TAT-miR122 VLPs labeled with FITC; d: amplification of single Hep3B cell only with FITC; e: amplification of single Hep3B cell only with DAPI; f: merged d and e. **Lower panel**, Penetrating cytomembrane of 2MS-TAT-miR122 VLPs in Huh7 cell. a, untreated cells; b, Huh7 treated with MS-miR122 VLPs labeled with FITC; c: Huh7 treated with 2MS-TAT-miR122 VLPs labeled with FITC; d: amplification of single Huh7 cell only with FITC; e: amplification of single Huh7 cell only with DAPI; f: merged d and e. **B.** The relative expression level of miR-122 in different cell lines treated with different concentration VLPs. 2MS-TAT-miRNC VLPs (40μg/mL), low concentration of 2MS-TAT-miR122 VLPs (8μg/mL) and high concentration of 2MS-TAT-miR122 VLPs (40μg/mL) treated with Hep3B, HepG2, Huh7, and Hela cell lines. The relative expression level of miR122 was analyzed by RT-qPCR (n=3). **, P < 0.01; *, P < 0.05. **C.** The relative expression level of miR122 at different time after treated with 2MS-TAT-miR122 VLPs in Hep3B cell lines. 0 h, 6h, 12 h, 24 h, 36 h, 48 h, 72 h, 96 h after treatment of 2MS-TAT-miRNC VLPs or 2MS-TAT-miR122 VLPs were analyzed (n=3). The relative expression level of miR-122 was analyzed by RT-qPCR. **, P < 0.01; *, P < 0.05.

To identify whether recombinant MS2 VLPs displaying TAT peptide could penetrate the cell membrane, the 2MS-TAT-miR122 VLPs and MS-miR122 VLPs labeled with fluorescein isothiocyanate (FITC, Sigma, USA) were respectively incubated with Hep3B and Huh7 cells. Blue fluorescence from 4′, 6-diamidino-2-phenylindole (DAPI) was observed in the nuclei; green fluorescence was emitted by FITC (Figure 3A).

After incubation with 2MS-TAT-miR122 VLPs labeled with FITC for 6 h, the green fluorescence was observed in the cytoplasm of both Hep3B (Figure 3, upper panel) and Huh7 cells (Figure 3, lower panel). By contrast, no green fluorescence could be detected in the cytoplasm with MS-miR122 VLPs labeled with FITC or in untreated groups, indicating that the TAT peptide was displayed successfully on the surface of 2MS-TAT-miR122 VLPs and could lead VLPs to penetrate membranes of various cells.

### Identification of delivery capability of 2MS-TAT-miR122 VLPs packed with miR-122

In order to identify whether 2MS-TAT-miR122 VLPs could induce expression of mature miR-122 in human cells, we transfected four different kinds of human cancer cell lines with 2MS-TAT-miR122 VLPs or 2MS-TAT-miRNC VLPs. The expression level of mature miR-122 was detected by real-time PCR. The low-level expression of endogenous miR-122 was detected in Hela, Hep3B, HepG2, and Huh7 cells (Figure 3B). After addition of 2MS-TAT-miR122 VLPs, high levels of miR-122 could be detected in the four cell lines, compared to the inconspicuous changes in 2MS-TAT-miRNC VLP treated groups. The dose-dependent response was analyzed with two doses of 2MS-TAT-miR122 VLPs in the four cell lines (Figure 3B). A dose-dependent change could be observed in all cells, and was most obvious in Hela and Hep3B cells.

Considering the inhibitory effects of miR-122 were mainly seen in hepatocarcinoma cell lines, Hep3B was selected for further time-lapse response detection. After transfection with 40 μg/mL 2MS-TAT-miR122 VLPs, Hep3B cells were harvested at 6, 12, 24, 36, 48, 72, and 96 h post-transfection. During the whole process, 24 h after transfection was the most significant time point for miR-122 detection, and the increase in miR-122 could also be detected during the course of the 96 h observed (Figure 3C).

### VLPs cytotoxity, inhibitory effects on proliferation, migration, and invasion in vitro

**Figure 4:**
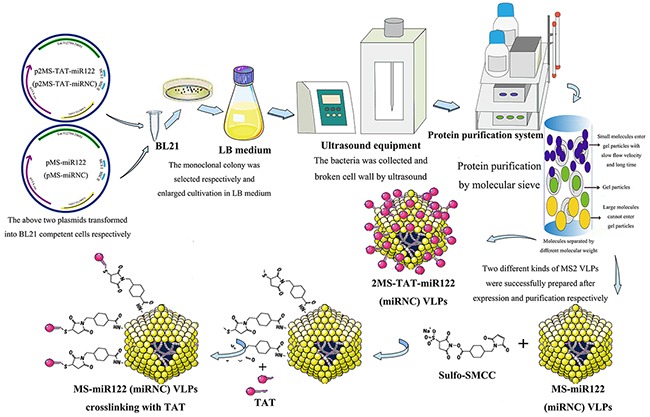
The flow chart of preparation for MS2 VLPs used in this research

To confirm the suppression of HCC by 2MS-TAT-miR122 VLPs, MS-miR122 VLPs and its negative control were prepared (Figure 4). When the expression and purification of MS-miR122 VLPs and MS-miRNC VLPs were completed, VLPs were identified using TEM, RT-PCR, nuclease-resistant test, and SDS-PAGE (Supplementary Figure S1). MS-miR122 VLPs and MS-miRNC VLPs were crosslinked with TAT peptide using crosslinker. The bands above the ordinary wild-type VLPs (14 kDa) indicated that the crosslinking was successful (Supplementary Figure S1E).

**Figure 5:**
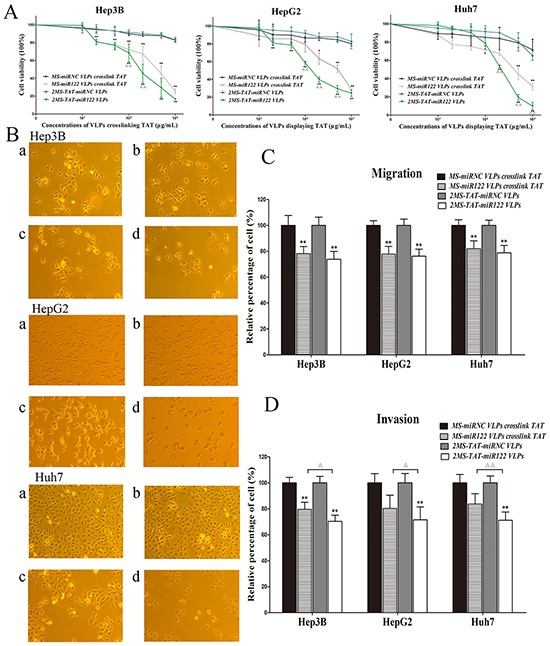
The inhibitory effects of 2MS-TAT-miR122 VLPs for proliferation, migration and invasion **A.** CCK-8 assay was performed to determine the proliferation of three types of hepatocellular carcinoma cell lines: Hep3B, HepG2 and Huh7 treated with 2MS-TAT-miR122 VLPs, 2MS-TAT-miRNC VLPs, MS-miR122 VLPs or MS-miRNC VLPs (n=3). **B.** Effects observation under light microscope of three types of hepatocellular carcinoma cell lines: Hep3B, HepG2 and Huh7 treated with different MS2 VLPs, a: MS-miRNC VLPs crosslinked with TAT; b: 2MS-TAT-miRNC VLPs; c: MS-miR122 VLPs crosslinked with TAT; d: 2MS-TAT-miR122 VLPs. **C.** Transwell cell migration assays performed with four kinds of MS2 VLPs (n=3). **D.** Transwell cell invasion assays performed with four kinds of MS2 VLPs (n=3). All the data above are represented as mean ± SD. Data compared with their negative controls is represented by: * P < 0.05, ** P < 0.01, whereas data from 2MS-TAT-miR122 VLPs treated group compared with MS-miR122 VLPs crosslinked with TAT treated group is represented by: Δ P < 0.05, ^ΔΔ^ P < 0.01.

To determine cellular toxicity of 2MS-TAT-miR122 VLPs, CCK8 assays were conducted. The results showed that the half maximal inhibitory concentration (IC50) of VLPs was > 100μg/mL in Hep3b, HepG2 and Huh7 (Figure 5A). Although the exact value of IC50 could not be confirmed, three kinds of cells viability under the conventional dose in this experiment (40 μg/mL) should be higher than 90%.

CCK8 assays were indicated the inhibitory effect of 2MS-TAT-miR122 VLPs on HCC proliferation simultaneously. Hep3B, HepG2, and Huh7 were treated with series concentrations of MS2 VLPs, and the results indicated that both 2MS-TAT-miR122 VLPs and MS-miR122 VLPs crosslinked with TAT could effectively inhibit proliferation in above cell lines. When the drug concentrations exceeded 50 μg/mL, more significant inhibitory effects (*P* < 0.05) could be observed in 2MS-TAT-miR122 VLPs treated groups, than in MS-miR122 VLPs crosslinked with TAT groups (Figure 5A). This was also visualized under a light microscope (Figure 5B). When treated with VLPs, cell counts of the positive treatment groups were significantly decreased in Hep3B, HepG2 and Huh7, especially in 2MS-TAT-miR122 VLPs treated groups.

To further examine the influence of 2MS-TAT-miR122 VLPs in neoplasm invasion and migration, we performed transwell migration and invasion assays. The results for migration revealed approximately 20% decrease rate in 2MS-TAT-miR122 VLPs treated groups (Figure 5C). Nevertheless, no visible difference between 2MS-TAT-miR122 VLPs and MS-miR122 VLPs crosslinked with TAT groups was observed (*P* > 0.05). In invasion tests for the three kinds of cells, the decrease for 2MS-TAT-miR122 VLPs treated groups was nearly 30%, whereas that for MS-miR122 VLPs crosslinked with TAT groups was 20% (Figure 5D), which indicated a statistically significant difference (*P* < 0.05) between the above two approaches.

### The conditions of cell apoptosis and target protein expression of miR-122

**Figure 6:**
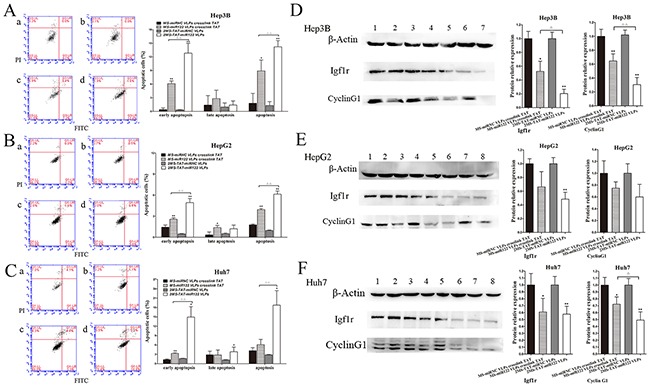
Cell apoptosis and target protein expression of miR-122 **A.** The apoptotic rates of Hep3B cells were detected by flow cytometry (n=3) treated with MS2 VLPs, a: MS-miRNC VLPs crosslinked with TAT; b: 2MS-TAT-miRNC VLPs; c: MS-miR122 VLPs crosslinked with TAT; d: 2MS-TAT-miR122 VLPs. **B.** The apoptotic rates of HepG2 cells were detected by flow cytometry (n=3) treated with MS2 VLPs, a: MS-miRNC VLPs crosslinked with TAT; b: 2MS-TAT-miRNC VLPs; c: MS-miR122 VLPs crosslinked with TAT; d: 2MS-TAT-miR122 VLPs. **C.** The apoptotic rates of Huh7 cells were detected by flow cytometry (n=3) treated with MS2 VLPs, a: MS-miRNC VLPs crosslinked with TAT; b: 2MS-TAT-miRNC VLPs; c: MS-miR122 VLPs crosslinked with TAT; d: 2MS-TAT-miR122 VLPs. **D.** Western blot analysis of Igf1r, cyclin G1 in Hep3B cell after treatment of different MS2 VLPs. β-Actin protein was used as an internal control. 1: pMS-miRNC VLPs crosslinked with TAT; 2: 2MS-TAT-miRNC VLPs; 3, 4: pMS-miR122 VLPs crosslinked with TAT; 5, 6: 2MS-TAT-miR122 VLPs. **E.** Western blot analysis of Igf1r, cyclin G1 in HepG2 cell after treatment of different MS2 VLPs. β-Actin protein was used as an internal control. 1: pMS-miRNC VLPs crosslinked with TAT; 2: 2MS-TAT-miRNC VLPs; 3, 4: pMS-miR122 VLPs crosslinked with TAT; 5, 6: 2MS-TAT-miR122 VLPs. **F.** Western blot analysis of Igf1r, cyclin G1 in Huh7 cell after treatment of different MS2 VLPs. β-Actin protein was used as an internal control. 1: pMS-miRNC VLPs crosslinked with TAT; 2: 2MS-TAT-miRNC VLPs; 3, 4: pMS-miR122 VLPs crosslinked with TAT; 5, 6: 2MS-TAT-miR122 VLPs. All the data above are represented as mean ± SD. Data compared with their negative controls is represented by: * P < 0.05, ** P < 0.01, whereas data from 2MS-TAT-miR122 VLPs treated group compared with MS-miR122 VLPs crosslinked with TAT treated group is represented by: ^Δ^ P < 0.05, ^Δ Δ^ P < 0.01.

Next, we focused on cell apoptosis, which also contributes to the inhibition of cancer cells. miR-122 is known to induce hepatoma cell apoptosis [19, 20, 41]. Therefore, we investigated whether the miR-122 harbored by MS2 VLP displaying TAT could also induce apoptosis in Hep3B, HepG2, and Huh7 cells. Both 2MS-TAT-miR122 VLPs and MS-miR122 VLPs crosslinked with TAT could induce apoptosis in the three mentioned cell lines, particularly in Hep3B and Huh7 cells (Fig 6A and C). Additionally, the apoptosis rate was more significant in 2MS-TAT-miR122 VLPs group compared to in MS-miR122 VLPs crosslinked with TAT groups (*P* < 0.01).

Cyclin G1 and Igf1r are the downstream target proteins of miR-122 which have been verified previously [13–15]. We selected the above two targets of miR-122 for further investigations. In the three cell lines, both cyclin G1 and Igf1r were down-regulated in 2MS-TAT-miR122 VLPs treated group. In Hep3B cells, suppression by 2MS-TAT-miR122 VLPs was more significant (*P* < 0.05) compared to that by MS-miR122 VLPs crosslinked with TAT (Figure 6D). Considering all the results of cytology experiments, we selected Hep3B cells, which reflected the most obvious inhibitory effects of 2MS-TAT-miR122 VLPs treatment, to establish animal models and perform subsequent experiments.

### Tumor suppression for 2MS-TAT-miR122 VLPs in vivo

**Figure 7:**
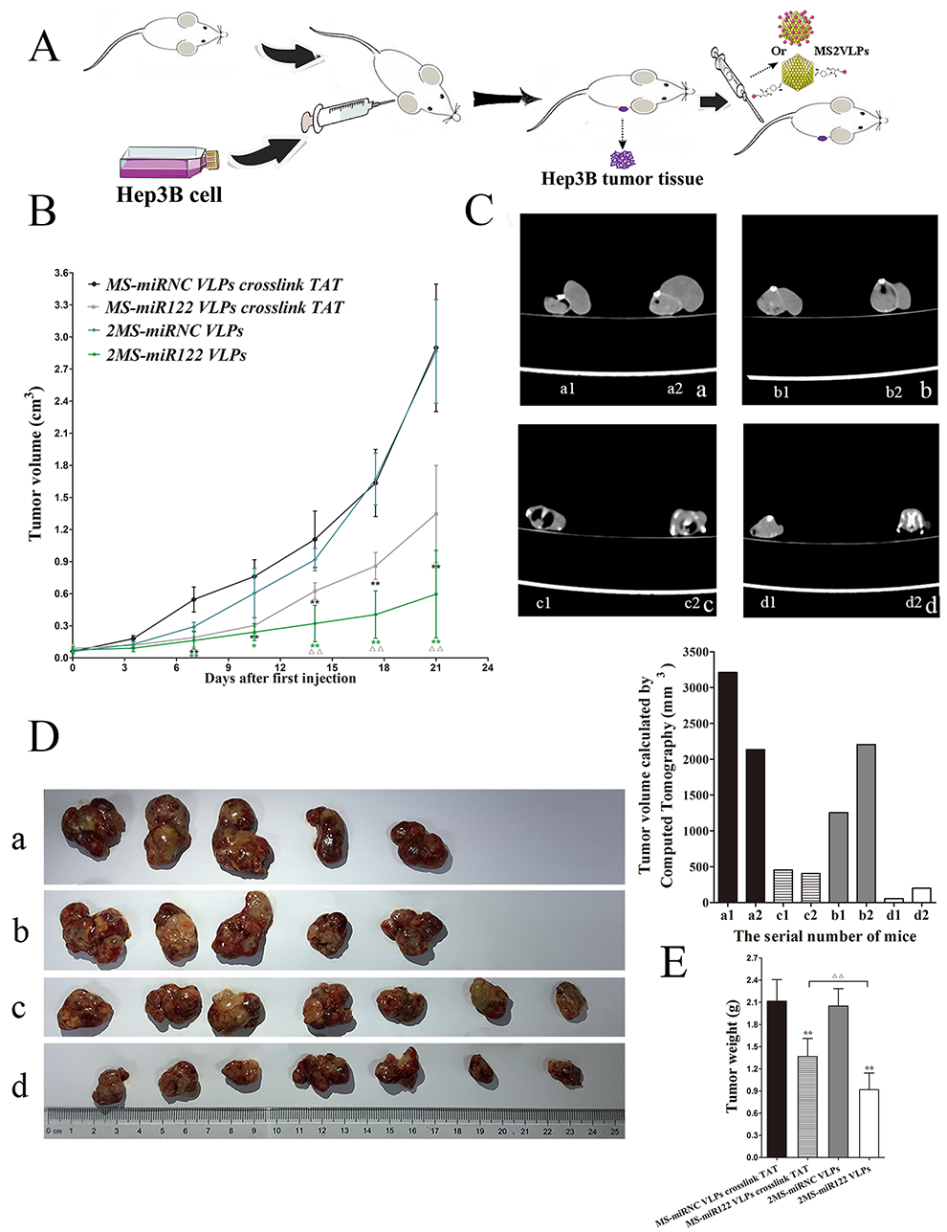
The effects on tumor growth in vivo after VLPs treatment **A.** The flow diagram of animal experiments. **B.** Tumor growth curve of different groups after treated with VLPs. **C.** CT photographs for two mice in each groups after treated with different VLPs, a: MS-miRNC VLPs crosslinked with TAT ; b: 2MS-TAT-miRNC VLPs; c: MS-miR122 VLPs crosslinked with TAT; d: 2MS-TAT-miR122 VLPs. **D.** Tumors were captured after three weeks' treatment of different VLPs, a: MS-miRNC VLPs crosslinked with TAT; b: 2MS-TAT-miRNC VLPs; c: MS-miR122 VLPs crosslinked with TAT; d: 2MS-TAT-miR122 VLPs. **E.** Tumor weights of different groups after treated with VLPs. Data compared with their negative controls is represented by: * P < 0.05, ** P < 0.01, whereas data from 2MS-TAT-miR122 VLPs treated group compared with MS-miR122 VLPs crosslinked with TAT treated group is represented by: ^Δ^ P < 0.05, ^Δ Δ^ P < 0.01.

In order to investigate the inhibitory effects of 2MS-TAT-miR122 VLPs in vivo, the nude mouse models of HCC were established. After inoculation of Hep3B subcutaneously, the tumor-burdened BALB/c nude mice were randomly assigned and administrated MS2 VLPs by tail vein (Figure 7A). The size of tumors was recorded before therapy and after each injection of VLPs. From tumor growth curve (Figure 7B), we could clearly learn that the growth speed of 2MS-TAT-miR122 VLPs treated group and MS-miR122 VLPs crosslinked with TAT treated group were both remarkably slower compared with their respective negative controls (*P* < 0.01). Moreover, the inhibitory effects of 2MS-TAT-miR122 VLPs was more significant (*P* < 0.01) than MS-miR122 VLPs crosslinked with TAT group, especially during the period of administration of the last three injections.

After the last injection was administered, two mice from each of the groups were selected to perform CT scan (Figure 7C). Tumor volume was calculated from the CT scan, which indicated smaller tumor sizes for 2MS-TAT-miR122 VLPs group, than its negative control and MS-miR122 VLPs crosslinked with TAT groups. Similarly, the final tumor mass (Figure 7D) and tumor weight (Figure 7E) of the 2MS-TAT-miR122 VLPs group was much less than other groups (*P* < 0.01).

### H&E staining, immunohistochemistry and western blot for animal tumors

**Figure 8:**
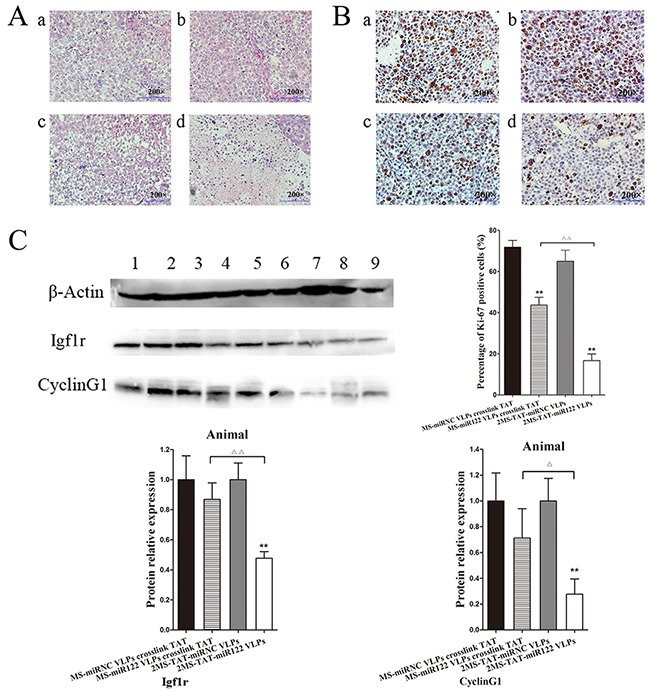
Inhibition effects of 2MS-TAT-miR122 VLPs after tumors captured from mice **A.** H&E staining of different groups after treated with VLPs, a: MS-miRNC VLPs crosslinked with TAT; b: 2MS-TAT-miRNC VLPs; c: MS-miR122 VLPs crosslinked with TAT; d: 2MS-TAT-miR122 VLPs. **B.** Immunohistochemistry with the Ki-67 of different groups after treated with VLPs, a: MS-miRNC VLPs crosslinked with TAT; b: 2MS-TAT-miRNC VLPs; c: MS-miR122 VLPs crosslinked with TAT; d: 2MS-TAT-miR122 VLPs. **C.** Western blot analysis of Igf1r, cyclin G1 for tumor tissues after treatment of different MS2 VLPs. β-Actin protein was used as an internal control. 1: pMS-miRNC VLPs crosslinked with TAT; 2: 2MS-TAT-miRNC VLPs; 3, 4, 5: pMS-miR122 VLPs crosslinked with TAT; 6, 7, 8: 2MS-TAT-miR122 VLPs. All the data above are represented as mean ± SD. Data compared with their negative controls is represented by: * P < 0.05, ** P < 0.01, whereas data from 2MS-TAT-miR122 VLPs treated group compared with MS-miR122 VLPs crosslinked with TAT treated group is represented by: ^Δ^ P < 0.05, ^Δ Δ^ P < 0.01.

H&E staining revealed degeneration and necrosis of tumor tissue in the group of 2MS-TAT-miR122 VLPs (Figure 8A). Immunohistochemistry with Ki-67, a nuclear protein associated with cell proliferation, was performed. Ki-67 staining of the 2MS-TAT-miR122 VLPs group was significantly lower than others (Figure 8B) (*P* < 0.01). The expression levels of cyclin G1 and Igf1r significantly decreased in 2MS-TAT-miR122 VLPs group (Figure 8C) (*P* < 0.01), which was in accordance with the in vitro results of the Hep3B cell line. Taken together, the results indicated that 2MS-TAT-miR122 VLPs could effective suppress tumor growth in vitro and in vivo.

## DISCUSSION

To realize the inhibitory effects on HCC, an effective delivery system for miR-122 was extraordinarily important. In a previous study, MS2 VLP based delivery system for microRNAs has been established and applied to SLE and osteoporosis therapy in our laboratory, which reflected satisfactory effects. However, a prominent issue with this transporter was the low efficiency of crosslinking of TAT peptides with MS2 VLP. High cost, long cycle, and rigorous preparation conditions were also inevitable problems that needed to be confronted.

With the purpose of improving the original MS2 VLP base delivery system, phage surface display technique was taken into consideration. According to previous reports, three different sites on the bacteriophage MS2 VLP allow for surface display of exogenous peptides: the C-terminus, AB-loop, and N-terminus. For C-terminus, less reports and inferior effects of exogenous peptide accommodation have been observed [35]. Several descriptions for the insertion of peptides on the AB-loop of MS2 have been confirmed successfully [36, 42], which may be benefit from the scattered locations on the surface of MS2 VLP of AB-loop in space. However, the tolerance for peptide insertion on the AB-loop was limited, ranging from 6-10 amino acids in general. The length of HIV TAT 47-57 peptide was slightly more than the capacity of AB-loop, hence N-terminus appeared appropriate to select. Moreover, a series of researches have demonstrated that the peptides could be successfully displayed on the N-terminus of MS2 [34–36], because the N-terminus of MS2 dimer coat protein could be modified to display peptides ranging from 10 to 27 amino acids without affecting VLP assembly. Under these circumstances, N-terminus was a better choice for TAT peptide insertion.

Fortunately, TAT peptide was successfully displayed on the surface of MS2 VLP with cell-penetrating ability, confirmed by confocal microscopy. The expression of miR-122 was clearly detected in various cells treated with 2MS-TAT-miR122 VLPs. Dose-dependent effects and the changes of expression over time were both reflected after treated with 2MS-TAT-miR122 VLPs.

Next, the inhibitory function of 2MS-TAT-miR122 VLPs in HCC was further tested. To better identify the anti-tumor effects, MS-miR122 VLPs conjugated with TAT were used for comparison. It was observed that the proliferation, migration and invasion was inhibited, the apoptosis was induced and the downstream target proteins were suppressed in human hepatoma cells treated with 2MS-TAT-miR122 VLPs, which was agreed with published previously results on miR-122. Encouragingly, there were more apparent suppression functions of 2MS-TAT-miR122 VLPs, than of MS-miR122 VLPs conjugated with TAT, particularly in Hep3B cells. In vivo assays, whether for tumor growth tendency, tumor weight, proliferative capability, or target protein expression, gave strong evidence of better inhibitory effects of 2MS-TAT-miR122 VLPs than others did.

Two reasons contributed to further analysis. For one, MS2 VLP displaying peptides was assembled with 90 copies of dimer of MS2 coat protein, which indicated that there might be 90 copies of TAT performing cell-penetrating functions on the surface of VLPs stably. However, the amount of conjugated TAT on wild-type MS2 VLPs was inconstant, and the crosslinking action was impressionable and unstable. Another reason was that the cell-penetrating peptide TAT has strong cell adherence, and enhances cellular uptake of the linked particles without dependence on receptors, temperature, or energy [43, 44]. Hence, the quantitative superiority of TAT peptides could increase efficacy of transmembrane and more obvious inhibitory effects of 2MS-TAT-miR122 VLPs.

However, the defects of this delivery system in HCC therapy should also be envisaged. One plausible drawback is that MS2 VLPs, as protein bio-macromolecules, could inevitably provoke an immune response, which would cause them to be cleared by macrophages or other immune cells, leading to reduction in their concentration and effectiveness. We detected high titers of anti-MS2 coat protein IgG antibody in the serum of mice after 3 weeks of therapy with the MS2 VLPs system. Although the immune response could reduce the treatment effect, HCC was also effectively controlled in tumor-bearing mice. Secondly no apparent adverse effects were observed in animal models. Furthermore, TAT was selected as a cell-penetrating peptide in this delivery system. Although the strong penetrating power of TAT has been proved, targeted delivery effect in HCC was relatively poor. Hence, a targeted MS2 VLP-based therapy system should be studied. Additionally, this delivery system was not suitable for HCC coupled with HCV infection. Because the increased level of miR-122 facilitated HCV replication, which could exert negative effects on HCC treatment eventually [45, 46].

Here, we have successfully established a novel miR-122 delivery system based on MS2 VLP surface displaying TAT peptide that can penetrate cytomembrane and inhibit HCC. Generally, this delivery system has significant advantages: (i) high biocompatibility and biodegradability of MS2 VLPs, (ii) feasible and simple preparation process, (iii) low cost of displaying TAT peptide without consideration of cross-linker or synthetic peptides, (iv) short preparation period, (v) stable productions without large inter-assay variations, (vi) suitable preparation conditions instead of rigorous requirements for PH value and ion concentrations, and (vii) more obvious effects for anti-tumor therapy than previously. Hence, recombinant MS2 VLPs displaying TAT are an efficient, feasible delivery strategy for miR-122 delivery and are promising in HCC treatment.

## MATERIALS AND METHODS

### Cell culture

The Hep3B, HepG2, Huh7, and HeLa cell lines were obtained from the Cell Bank of the Chinese Academy of Medical Science (Beijing, China). The Hep3B cell line was maintained in minimum essential medium (MEM, Gibco, Grand Island, NY), supplemented with 15% fetal bovine serum (FBS, Gibco, Grand Island, NY). The HepG2, Huh7, and Hela cell lines were cultured in Dulbecco's modified Eagle's medium (DMEM, Gibco, Grand Island, NY), supplemented with 10% FBS. All cell lines were cultured with 100 units/mL penicillin/streptomycin (Gibco, Grand Island, NY), and maintained in an incubator at 37°C with 5% CO_2_.

### Construction of plasmids

The sequence of pre-miR122 (negative control: non-coding pre-miRNA, pre-miRNC) (Table 1) was generated using artificial gene synthesis method, and inserted into the pACYCDuat-1 vector through NdeI/PacI sites (Sangon, Beijing, China) (Figure 1, bottom), then named pACYC-miR122 (negative control: pACYC-miRNC). HIV TAT 47-57 peptide sequence (C-47YGRKKRRQRRR-57) and a linker sequence (GGGGS) were combined, and added to the amino-terminus of dimer of the MS2 coat protein (Figure 1, top). The above sequences were generated through gene synthesis, and cloned into the plasmid pACYC-miR122 (negative control: pACYC-miRNC) using NcoI/HindIII sites, then named p2MS-TAT-miR122 (negative control: p2MS-TAT-miRNC) (Figure 1). The single coat protein sequence of MS2 bacteriophage was obtained from pESC-URA-MS2 (archived in our laboratory), and inserted into pACYC-miR122 (negative control: pACYC-miRNC) using NcoI/Hind III sites, then named pMS-miR122 (negative control: pMS-miRNC). All the primers required above are listed in Table 1.

**Table 1:** Plasmids, VLPs, primers and synthesized oligonucleotides used in this study

Materials	Characteristics
**Plasmids**	
pACYCDuat-1	Two target genes co-expression, T7 promoter, lac operator, ribosome binding site, T7 terminator, His Tag, S Tag, Chloramphenicol resistance gene
pACYC-miR122	pACYCDuat-1with pre-miR122 inserted in MCS2
pACYC-miRNC	pACYCDuat-1with pre-miRNC inserted in MCS2
p2MS-TAT-miR122	pACYC-miR122 with sequences of HIV TAT (47-57) with a linker combined with the amino-terminus of a dimer of MS2 coat protein inserted in MCS1
p2MS-TAT-miRNC	pACYC-miRNC with sequences of HIV TAT (47-57) with a linker combined with the amino-terminus of a dimer of MS2 coat protein inserted in MCS1
pESC-URA-MS2	pESC-URA with MS2 capsid gene (GenBank: GQ456167.1) inserted in MCS1 preserved by our laboratory
pMS-miR122	pACYC-miR122 with sequences of MS2 capsid gene cloned form pESC-URA-MS2 inserted in MSC1
pMS-miRNC	pACYC-miRNC with sequences of MS2 capsid gene cloned form pESC-URA-MS2 inserted in MSC1
**VLPs**	
2MS-TAT-miR122	Expression products of p2MS-TAT-miR122
2MS-TAT-miRNC	Expression products of p2MS-TAT-miRNC
MS-miR122	Expression products of pMS-miR122
MS-miRNC	Expression products of pMS-miRNC
**Primers and synthesized oligonucleotides**	
P122*	GGAATTCCATATG**ACATGAGGATCACCCATGT**CCTTAGCAGAGCTGTGGA GTGTGACAATGGTGTTTGTGTCTAAACTATCAAACGCCATTATCACACTAAATAGCTACTGCTAGGC**ACATGAGGATCACCCA TGT**TTAATTAAGG
PNC*	GGAATTCCATATG**ACATGAGGATCACCCATGT**CTGCAGAAGGTCACCCAG GGTAACGTTGACCTTGGTGTTGCTCTAGCAGCGATCGGTCATTGACTGCTC GAGCCAGGTCGACAGC**ACATGAGGATCACCC ATGT**TTAATTAAGG
pMS-F*	CATGCCATGGCTTCTAACTTTACTCAGTTCG
pMS-R*	CCCAAGCTTATGGCCGGCGTCT
p2MS-TAT-F*	CATGCCATGGCTTATGGTCGTAAAAAACG
p2MS-TAT-R*	CCCAAGCTTATGGCCGGCGTCT
U6 F	GCTTCGGCAGCACATATACTAAAAT
U6 R	CGCTTCACGAATTTGCGTGTCAT
miR-122 F	GGCTGGAGTGTGACAATGGT
miR-122 R	GTGCAGGGTCCGAGGT
miR-122 stem-loop	GTCGTATCCAGTGCAGGGTCCGAGGTATTCGCACTGGATACGACCAAACA
miR-122(miRNC) RT-F	CATATGACATGAGGATC
miR-122(miRNC) RT-R	GGGCTTAATTAAACATG

Note: *Sequences under the line are restriction enzyme recognition site. Sequences in bold are the pac site sequences.

### Preparation and verification of MS2 VLPs

The plasmid p2MS-TAT-miR122 and p2MS-TAT-miRNC were transformed into competent cells of the E. coli BL21 (DE3) strain respectively. The monoclonal colony was selected, amplified, harvested and wall-cracked. The proteins were purified and the products were harvested (Figure 2A). All of the preparation process was according to previous protocols [31], and the products were named 2MS-TAT-miR122 VLPs and 2MS-TAT-miRNC VLPs, respectively.

The prepared MS2 VLPs were verified by TEM (JEM-1230; JEOL, Tokyo, Japan), SDS-PAGE and western blot analysis with anti-MS2 antibody. RT-PCR and 1% agarose gel electrophoresis were performed as well. Additionally, after digested with 1μl DNase I (10 U/mL) and 1μl RNase A (10 mg/mL) at 37°C for 2 h, the products were analyzed by 1% agarose gel electrophoresis GelRed (Biotium, Hayward, CA) staining.

The prepared products of pMS-miR122 and pMS-miRNC plasmids were named MS-miR122 VLPs and MS-miRNC VLPs, respectively. These VLPs were conjugated to HIV TAT (47-57) peptides with a linker (Chinese Peptide Company, China). The crosslinking reagent sulfosuccinimidyl 4-(N-maleimidomethyl) cyclohexane-1-carboxylate (Sulfo-SMCC) was acquired from Thermo Fisher Scientific (Rockford, IL). SDS-PAGE was performed to verify the effect of crosslinking (Supplementary Figure S1).

### Preparation and analysis of samples by confocal fluorescence microscopy

The FITC (Sigma, St Louis, MO) was weighed and dissolved into dimethyl sulfoxide (DMSO). The 2MS-TAT-miR122 VLPs and MS-miR122 VLPs were crosslinked with dissolved FITC at 4°C for 2 h, separately. Hep3B and Huh7 cells were used in this experiment, and the process for sample preparation was according to the reported protocols [47]. Untreated cells and cells treated with MS-miR122 VLPs with FITC were set as negative controls. The Hep3B and Huh7 cell slides were analyzed with a confocal fluorescence microscope (Nikon A1, Tokyo, Japan).

### Quantitative real-time PCR

5 × 10^3^ cells were cultured in each well of a 24-well plate, and 2MS-TAT-miR122 VLPs or 2MS-TAT-miRNC VLPs were added to the wells with: (a) different concentrations of MS2 VLPs (8 or 40 μg/mL) for 24 h; (b) various cell lines (HeLa, Hep3B, HepG2 and Huh7) for 24 h; (c) final concentrations of 40 μg/mL MS2 VLPs for different time periods (6, 12, 24, 36, 48, 72 and 96 h) in Hep3B. The processes for RNA extraction, amplification and detection were according to previous protocols [48]. All reactions were run in triplicates, and U6 RNA was chosen as internal control. The qPCR results were analyzed relative to the Ct (threshold cycle) value, and converted to fold-change values according to the rules of 2^−ΔΔCt^ method. Primers are listed in Table 1.

### In vitro cytotoxity and proliferation assays

Cell proliferation and cytotoxity were monitored using Cell Counting Kit-8 assay (CCK-8, Dojindo, Japan). Cells (1 × 10^3^ per well) were cultured in 96-well plates and incubated at 37°C overnight. The HCC cell lines Hep3B, HepG2, and Huh7 were used in this experiment. 2MS-TAT-miR122 VLPs (negative control: 2MS-TAT-miRNC VLPs) or MS-miR122 VLPs (negative control: MS-miRNC VLPs) (100 μL each) of different concentrations (1 μg/mL to 1000 μg/mL) were respectively administered to different cell lines in 96-well plates for 72 h. CCK-8 reagent was added, and the absorbance at 450 nm was measured. All experiments were repeated three times.

### Observation under light microscope, and migration and invasion assays

Hep3B, HepG2 and Huh7 cells were seeded in 6-well plates (5 × 10^5^ cells per well), and treated with 40 μg/mL 2MS-TAT-miR122 VLPs (negative control: 2MS-TAT-miRNC VLPs) or MS-miR122 VLPs (negative control: MS-miRNC VLPs) crosslinked with TAT, individually for 36 h. The conditions of different treated cells were photographed with a light microscope (Leica DMIL-LED, German).

Subsequently, the migration and invasion assays were performed using Transwell chambers, with or without a Matrigel (BD Biosciences, Franklin Lakes, NJ) coating. After incubation for 24 hours, the cells on the upper surface of the filters were removed with a cotton swab. The cells that had migrated or invaded into the lower surface of the filters were fixed and stained with a Diff-Quik Staining Kit (ZKKA Biotechnology, Beijing, China), and were counted in five random 400× visual fields under a microscope for each group.

### Cell apoptosis assay

Hep3B, HepG2 and Huh7 cells were treated with 40 μg/mL 2MS-TAT-miR122 VLPs (negative control: 2MS-TAT-miRNC VLPs) or MS-miR122 VLPs (negative control: MS-miRNC VLPs) crosslinked with TAT, individually for 48 h. Then the cells treated for apoptosis analysis were digested, washed twice with phosphate-buffered saline (PBS, pH 7.4), double-stained with Annexin V-FITC and propidium iodide (PI, BD Biosciences, Franklin Lakes, NJ), and analyzed with a flow cytometer (BD Accuri C6, Franklin Lakes, NJ). The percentage of early and late apoptotic cells was distinguished, counted, and compared between different treatment groups. All experiments were performed in triplicate.

### Western blot assay

Hep3B, HepG2 and Huh7 cells were treated with 40 μg/mL 2MS-TAT-miR122 VLPs (negative control: 2MS-TAT-miRNC VLPs) or MS-miR122 VLPs (negative control: MS-miRNC VLPs) crosslinked with TAT, individually for 48 h. Western blot was conducted as previously described [33], using primary antibodies at 1:1000 dilution against the following proteins : Igf1r (Cell Signaling Technology, Boston, MA), cyclin G1 (Santa Cruz, Dallas, TX), β-Actin (Santa Cruz) and MS2 coat protein (Millipore, Boston, MA).

### Tumor models establishment and VLPs injection

Female BALB/c nude mice (3–4 weeks old, specific pathogen-free/viral antibody-free, SPF/VAF) were purchased from Vital River (Beijing, China), and fed a standard diet, under the conditions of SPF isolation in the Institute of Laboratory Animal Science (Beijing, China). All animals used in this experiment were approved by the Ethics Committee of the National Center for Clinical Laboratories. Hep3B cells (1 × 10^7^), combined with BD Matrigel Basement Membrane Matrix (BD, Franklin Lakes, NJ), were subcutaneously injected into the posterior flank of each mouse. When the tumor reached approximately 0.5 cm^3^, observed with naked eye, the mice were ready for VLP administration, after being randomly grouped. Four groups were designed in this experiment: (i) treated with 2MS-TAT-miRNC VLPs (5 mice), (ii) treated with MS-miRNC VLPs crosslinked with TAT (5 mice), (iii) treated with 2MS-TAT-miR122 VLPs (7 mice), and (iv) treated with MS-miR122 VLPs crosslinked with TAT (7 mice). VLPs (100 ng per administration) were administrated twice a week via tail vein for 3 weeks. Tumor volumes were calculated using the equation,

$$\text{V}=0.5\times \text{a}\times {\text{b}}^{2}$$

where *V* is volume, *a* is longitudinal diameter and *b* is latitudinal diameter.

### CT scan and tumor preservation

After the last dose was administered, two mice from each treatment group were selected for CT scan (GE discovery 750, Beijing Hospital, Beijing, China) and the tumor volume for each mouse was calculated in the way of CT scan. Subsequently, all tumor-bearing mice were euthanized. The tumors were separated and weighed, and the subcutaneous growth of each tumor was recorded.

### Hematoxylin and eosin (H&E) staining, and immunohistochemistry

The isolated tumor tissues were fixed with 4% paraformaldehyde, embedded in paraffin, and sectioned to 5 μm thickness. HE staining was followed using previous protocols [49], and immunohistochemistry was performed with the primary antibody Ki67 (Abcam, Cambridge, UK) at 1:1000 and HRP-conjugated secondary antibodies (ZSGB-Bio, Beijing, China). The experiment was conducted as previously described [49, 50].

### Statistical analysis

Statistical analyses were performed using SPSS 20.0 software, and presented with the GraphPad Prism 6 Software. The differences between the groups were tested using two-tailed Student's *t*-test. The results were considered to be statistically significant if *P* < 0.05. Additionally, data compared with their negative controls is represented by: * *P* < 0.05, ** *P* < 0.01, whereas data from 2MS-TAT-miR122 VLPs treated group compared with MS-miR122 VLPs crosslinked with TAT treated group is represented by: ^*^ *P* < 0.05, ^**^ *P* < 0.01.

## SUPPLEMENTARY FIGURE


