# Supplementary material for: Novel miR-122 delivery system based on MS2 virus like particle surface displaying cell-penetrating peptide TAT for hepatocellular carcinoma
Source: Oncotarget. 2016 Jul 18;7(37):59402–16. doi: 10.18632/oncotarget.10681 (PMC5312320; doi:10.18632/oncotarget.10681)
Supplement: Supplementary file 1 [file oncotarget-07-59402-s001.pdf]

Novel miR-122 delivery system based on MS2 virus like particle surface displaying cell-penetrating peptide TAT for hepatocellular carcinoma

SUPPLEMENTARY FIGURE

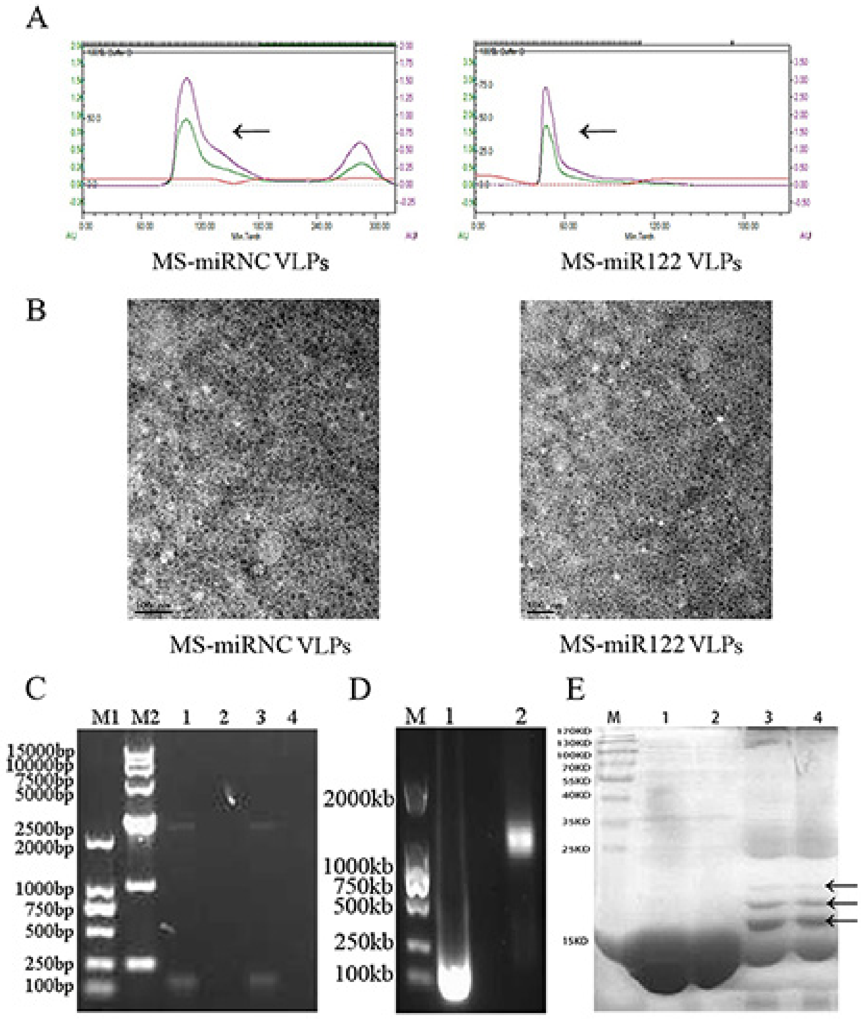

Supplementary Figure S1: Identification of MS-miR122 VLPs and its negative control. A. Purification of VLPs. The peak of the target protein is marked by an arrow. Left panel, MS-miRNC VLPs; right panel, MS-miR122 VLPs. **B.** Verification of VLPs by TEM. Left panel, MS-miRNC VLPs; Right panel, MS-miR122 VLPs. **C.** RT-PCR detection of miRNA packaged by the VLPs. Lane M1, DL2000; Lane M2, DL15000; Lane 1, MS-miRNC VLPs; Lane 2, negative control; Lane 3, MS-miR122 VLPs; Lanes 4, negative control. **D.** Nuclease resistance assay of VLPs. The MS2 VLPs in Lane 2 was incubated with DNase I and RNase A, but those in Lanes 1 were not. **E.** Verification of VLPs by SDS-PAGE. Lane M, molecular mass marker; Lane 1, MS-miRNC VLPs; Lane 2, MS-miR122 VLPs; Lane 3, MS-miRNC VLPs crosslinking TAT; Lane 4, MS-miR122 VLPs crosslinking TAT. The target protein is marked by an arrow.
